# Investigating the Effectiveness of Different Porous Nanoparticles as Drug Carriers for Retaining the Photostability of Pinosylvin Derivative

**DOI:** 10.3390/pharmaceutics16020276

**Published:** 2024-02-15

**Authors:** Fadak Howaili, Atefeh Saadabadi, Ermei Mäkilä, Ekaterina Korotkova, Patrik C. Eklund, Outi M. H. Salo-Ahen, Jessica M. Rosenholm

**Affiliations:** 1 Pharmaceutical Sciences Laboratory, Faculty of Science and Engineering, Åbo Akademi University, 20500 Turku, Finland; fadak.howaili@abo.fi (F.H.); atefeh.saadabadi@abo.fi (A.S.); osalo@abo.fi (O.M.H.S.-A.); 2 Laboratory of Molecular Science and Engineering, Faculty of Science and Engineering, Åbo Akademi University, 20500 Turku, Finland; patrik.c.eklund@abo.fi; 3 Laboratory of Industrial Physics, Department of Physics and Astronomy, University of Turku, 20014 Turku, Finland; emmaki@utu.fi; 4 Laboratory of Natural Materials Technology, Faculty of Science and Engineering, Åbo Akademi University, 20500 Turku, Finland; ekaterina.korotkova@abo.fi; 5 Structural Bioinformatics Laboratory, Faculty of Science and Engineering, Åbo Akademi University, 20500 Turku, Finland

**Keywords:** gas chromatography, photostability, pinosylvin monomethyl ether, porous nanoparticles, drug nanocarriers

## Abstract

Pinosylvin monomethyl ether (PsMME) is a natural compound known for its valuable bioactive properties, including antioxidant and anti-inflammatory effects. However, PsMME’s susceptibility to photodegradation upon exposure to ultraviolet (UV) radiation poses a significant limitation to its applications in the pharmaceutical field. This study, for the first time, introduces a strategy to enhance the photostability of PsMME by employing various nanoformulations. We utilized mesoporous silica nanoparticles (MSNs) coated with polydopamine via a poly(ethylene imine) layer (PDA–PEI–MSNs), thermally carbonized porous silicon nanoparticles (TCPSi), and pure mesoporous polydopamine nanoparticles (MPDA). All these nanocarriers exhibit unique characteristics, including the potential for shielding the drug from UV light, which makes them promising for enhancing the photostability of loaded drugs. Here, these three nanoparticles were synthesized and their morphological and physicochemical properties, including size and ζ-potential, were characterized. They were subsequently loaded with PsMME, and the release profiles and kinetics of all three nanoformulations were determined. To assess their photoprotection ability, we employed gas chromatography with a flame ionization detector (GC-FID) and gas chromatography–mass spectrometry (GC-MS) to assess the recovery percentage of loaded PsMME before and after UV exposure for each nanoformulation. Our findings reveal that MPDA exhibits the highest protection ability, with a remarkable 90% protection against UV light on average. This positions MPDA as an ideal carrier for PsMME, and by extension, potentially for other photolabile drugs as well. As a final confirmation of its suitability as a drug nanocarrier, we conducted cytotoxicity evaluations of PsMME-loaded MPDA, demonstrating dose-dependent drug toxicity for this formulation.

## 1. Introduction

Pinosylvin and its derivatives are naturally occurring stilbenoid compounds found in spruce and pine trees, and have garnered considerable attention due to their diverse range of biological and pharmaceutical activities, encompassing antimicrobial, anti-inflammatory, anticancer, neuroprotective, and anti-allergic effects [1]. While the anti-inflammatory impact of stilbenoid compounds bears resemblance to that of established commercial anti-inflammatory agents [2], their physicochemical properties limit their therapeutic potential. PsMME and stilbenoids, in general, typically feature long hydrocarbon chains. As a rule, compounds with such hydrocarbon chains often exhibit low solubility in water [3]. Compromised aqueous solubility and the resulting poor bioavailability, coupled with heightened susceptibility to external influences, such as air, UV light, and oxidative enzymes, present substantial challenges [3]. UV light exposure, in particular, triggers polymerization and cis–trans isomerization of stilbenoids [4], thus diminishing their stability and efficacy. To address these limitations and unlock the full therapeutic capabilities of stilbenoids, there is a crucial need to tailor the physicochemical attributes of these compounds. This endeavor is aimed at maintaining therapeutic doses of stilbenoids at a safe level, by simultaneously ensuring their high stability and suitable solubility.

A recent study has indicated that enabling formulations, especially through the encorporation of stilbenoids, can augment their therapeutic outcomes [5]. Therefore, our study utilizes versatile nanocarriers: three different types of mesoporous nanoparticles, to modulate the physicochemical properties of pinosylvin monomethyl ether (PsMME). Mesoporous silica nanoparticles (MSNs) have long been used as carriers to effectively increase the solubility and dissolution rates of diverse drugs and biologically active agents [6]. Furthermore, the inorganic silica matrix of MSNs offers a platform for stabilizing the incorporated agents during storage, thereby extending the shelf life of the loaded drug [7]. Given that amorphous silica is optically transparent, an effective approach to enhance the photostability of incorporated agents involves coating MSNs with polydopamine (PDA), a well-known UV-shielding material that helps preserve the biological activity of photolabile substances [8]. As a follow-up to this system, mesoporous polydopamine (MPDA) nanoparticles composed only of PDA, i.e., without the MSN skeleton, have also gained prominence, leveraging the simplicity and biocompatibility of PDA [9]. However, the properties of this system are not as flexibly controllable as in the case of MSNs.

Moreover, porous silicon (PSi) materials, also known for their stabilizing matrix, biocompatibility, biodegradability, and enhanced drug permeability properties, have emerged as valuable tools for a variety of biomedical applications [10]. Distinguished from the bottom-up fabrication method of mesoporous silica materials, PSi materials are created through top-down etching and milling techniques. Their distinct optical properties, including the ability to absorb UV-light across various wavelengths due to their unique porous structure, position them as ideal candidates for further exploration as carriers of photolabile drug compounds [11,12]. The surface chemistry of PSi is also easily modifiable, such as through thermal carbonization with acetylene (TCPSi), providing the nanocarrier with high aqueous stability [13] (contrary to their silica counterparts).

Introduction of dark color components into nano-systems has been documented to enhance the saturation of structural colors by effectively absorbing scattered light across a broad wavelength spectrum [14]. In the current study, we conducted a comprehensive evaluation of three distinct dark nanoparticles, namely PDA–PEI–MSNs [PEI = poly(ethylene imine)], MPDA, and TCPSi, with the aim of exploring their potential as nanocarriers for augmenting the photostability of PsMME as model drug. First, by employing techniques such as dynamic light scattering (DLS) and transmission electron microscopy (TEM), we characterized the physicochemical attributes of the nanoformulations. Subsequently, we assessed their drug loading degree (DL%), drug release profiles, and photostability, allowing for a systematic comparison of the nanoparticles’ respective performances. Notably, due to the superior photostability-conveying properties exhibited by MPDA, these nanoparticles were also assessed for their cytotoxicity with and without loaded drug to determine their cytocompatibility as a basis for further studies.

In summary, this study sets the stage for a detailed exploration into the potential of tailored nanocarriers to enhance the therapeutic viability of PsMME and other photolabile drugs, marking a significant stride toward overcoming its inherent limitations and maximizing its pharmaceutical potential. 

## 2. Materials and Methods

### 2.1. Reagents and Materials

The research utilized various materials and reagents, encompassing the following: tetraethyl orthosilicate (TEOS), cetyltrimethylammonium chloride solution (CTAC), triethylamine (TEA), (3-aminopropyl) triethoxysilane (APTES), decahydronaphthalene, ammonium nitrate, glacial acetic acid (100%), 4-(2-hydroxyethyl)-1-piperazineethanesulfonic acid (HEPES buffer) (25 mM, pH 7.4), dopamine hydrochloride, 1,3,5-trimethylbenzene (TMB, AR, 97%), pluronic F-127 (F127), methanol, acetone (HPLC grade), all internal standards, and ammonium persulfate. All these materials and reagents were commercially available and supplied by Sigma-Aldrich (Burlington, MA, USA). Ethanol (EtOH, 99.5%) and pyridine were sourced from VWR, while aziridine was supplied by Menadiona. PsMME (purity > 95%) was extracted at the Laboratory of Organic Chemistry at Åbo Akademi University (Figure S1) [15]. The silylation reagents hexamethyldisiloxane (HMDS) and trimethylchlorosilane (TMCS) were purchased from Aldrich Chemicals (St. Louis, MO, USA) and bis(trimethylsilyl)tri-fluoroacetamide (BSTFA) was obtained from Acros Organics (Geel, Belgium). Betulinol was isolated from birch bark and purified (purity > 99.6% confirmed by GC and GC-MS) at the Laboratory of Natural Materials Technology at the Åbo Akademi University [16]. Furthermore, Dulbecco’s Modified Eagle Medium (DMEM), penicillin–streptomycin, Dulbecco’s Phosphate Buffered Saline, and L-glutamine were all obtained from Lonza (Basel, Switzerland). Gibco fetal bovine serum (FBS) and MEM Non-Essential Amino Acids Solution (100×) (NEAA) were purchased from ThermoFisher (Waltham, MA, USA). Human embryonic kidney cells (HEK293) were generously provided by Prof. Hongbo Zhang’s research group at the Pharmaceutical Sciences Laboratory, Åbo Akademi University.

### 2.2. Synthesis of Nanoformulations 

#### 2.2.1. Synthesis of PDA–PEI–MSNs 

The synthesis of MSNs with 70 nm size and pore size of 5.5 nm was realized through the utilization of a biphasic stratification technique, as reported previously [17]. In a 100 mL flask, a mixture comprising 36 mL of water, 24 mL of CTAC as a template (10 wt%), and 0.18 g of triethylamine (TEA) as a catalyst was subjected to stirring within an oil bath maintained at 60 °C. After 1 h of stirring, a solution containing 20 mL of TEOS in decahydronaphthalene (20 *v*/*v*%) was gradually introduced and kept at 60 °C for 8 h. Subsequently, the resultant particles were collected through centrifugation of the reaction mixture at 18,000 rpm for 20 min at 10 °C. After discarding the supernatant, the particles underwent two washes with absolute EtOH, 99.5% under identical conditions. The removal of surfactant from the pores employed the ion exchange method, involving stirring with an ethanolic ammonium nitrate solution (0.6 wt%) at 60 °C for 6 h, followed by centrifugation for collection, a process repeated twice. Finally, the particles were rinsed twice with EtOH, 99.5% and stored in EtOH, 99.5% for subsequent utilization. The functionalization of the MSN surfaces involved the surface-hyperbranching polymerization of aziridine to form poly (ethylene imine) (PEI) as the first step. This strategic modification facilitated the subsequent coating of the MSNs with PDA, and it was performed as previously detailed by our research group [18]. PDA grafting onto the surfaces of PEI-MSNs was performed by oxidant-induced surface self-polymerization of dopamine hydrochloride. Briefly, a standard procedure was employed wherein 20 mg of PEI-MSNs was suspended in 10 mL of HEPES buffer at a concentration of 25 mM and a pH value of 7.4. In this suspension, 4 mg of dopamine hydrochloride was introduced, followed by 5 min of sonication to ensure thorough mixing. Subsequently, 4.8 mg of ammonium persulfate was added to the reaction mixture, serving as the initiator for the polymerization process. This reaction mixture was allowed to undergo polymerization overnight. Following completion of the reaction time, the resultant nanoparticles were separated by Scientific™ Sorvall LYNX 4000 centrifuge (Waltham, MA, USA) at 18,000 rpm for 20 min and subjected to a triple-phase washing with EtOH to eliminate any residual impurities [8].

#### 2.2.2. Synthesis of MPDA Nanoparticles

The preparation of MPDA was conducted using a one-pot synthesis approach, following the methods outlined in the existing literature [9]. F127 and TMB were dissolved in a mixture of deionized water and EtOH, 99.5% with the molar ratio of F127:112 TMB:156,067 deionized water:38,577 EtOH, 99.5% and placed in the stirrer. After 30 min of stirring, once a homogenous solution was achieved, 40 mg of TRIS was dissolved in deionized water and subsequently combined with the solution. Then, dopamine hydrochloride was added in the molar ratio of 28:12 TRIS to dopamine hydrochloride. After 24 h of continuous stirring, the resulting nanoparticles were isolated through centrifugation by Scientific™ Sorvall LYNX 4000 centrifuge at 18,000 rpm for 20 min. The product was washed, and the template was removed with a mixture of EtOH, 99.5% and acetone (2:1 *v*/*v*) three times for 30 min of sonication. The purified product was suspended in EtOH, 99.5% for further use. 

### 2.3. Fabrication of TCPSi Nanoparticles 

TCPSi nanoparticles were fabricated adapting a method described previously [19]. Briefly, monocrystalline boron-doped Si (100) wafers of 0.01–0.02 Ωcm resistivity were electrochemically anodized in 1:1 (vol.) HF (38%)-EtOH solution. The etching profile consisted of successive low and high current density pulses (50 mA/cm^2^ and 200 mA/cm^2^) for creating perforating high porosity layers to facilitate the fragmentation of the obtained free-standing multilayer PSi films. The PSi films were then thermally carbonized by inserting them into quartz tube under a continuous N_2_ flow. Initially, the films were treated under a 1:1 (vol.) flow of N_2_ and acetylene (C_2_H_2_) at room temperature for 15 min, followed by a 15 min heat treatment at 500 °C. The films were then allowed to cool back to room temperature under N_2_ flow. Finally, the films were again placed under a 1:1 N_2_-C_2_H_2_ flow at room temperature for 10 min, followed by thermal annealing for 10 min at 820 °C under N_2_ flow. The obtained TCPSi multilayer films were milled in EtOH in a ZrO_2_ grinding jar with ZrO_2_ grinding balls until reduced into nanoparticles using a high energy Fritsch Pulveristte 7 classic ball mill (Idar-Oberstein, Germany). The final particle size selection was carried out with centrifugation at 400 rpms for 8 h with 10 min intervals. 

### 2.4. Drug Incorporation into Nanoparticles

The loading of PsMME into the PEI–PDA–MSNs, MPDA, and TCPSi nanoparticles was achieved utilizing the solvent evaporation technique. Methanol serves as the solvent for PsMME in various scholarly investigations as a good solvent for the drug [20], which is required to reach high enough concentrations to enable utilization of the solvent evaporate ion method. Thus, 200 µg PsMME was dissolved in 300 μL of methanol, and then mixed with 1 mg nanoparticles using a spatula until it appeared dry. The intended drug loading degree was chosen to be within the detection range of the photostability measurements. Subsequently, the samples were allowed to air dry under hood for 48 h for further removal of solvent remnants. Following the drying process, the products were kept at +4 °C for subsequent experiments. The encapsulation efficiency (EE%) and drug loading (DL%) of nanoparticles were determined by leaching the PsMME from the nanoparticles. Briefly, 0.5 mg of the three types of PsMME-loaded nanoparticles were dispersed in methanol (*n* = 3) utilizing a bath sonicator for 15 min combined with vortex until well-dispersed solutions were obtained. Then, the nanoparticles were centrifuged for 20 min at 13,500 rpm. The supernatant was collected, and the amount of PsMME was estimated using the NanoDrop™ 2000c spectrophotometer (Thermo Scientific) at 300 nm. The EE% and DL% were calculated using the following Equations (1) and (2) [21,22].

(1) $$EE\%=\frac{Total\;amount\;of\;fed\;PsMME-free\;PsMME}{Total\;amount\;of\;PsMME}*100$$

(2) $$DL\%=\frac{Total\;amount\;of\;fed\;PsMME-free\;PsMME}{Weight\;of\;nanoparticles}*100$$

### 2.5. Physicochemical Characterization of Nanoparticles before and after PsMME Loading 

The hydrodynamic particle size, net surface charge, and polydispersity index (PDI) of MSN (including MSNs, PEI–MSNs, PEI–PDA–MSNs), MPDA, and TCPSi suspensions were assessed before and after loading of PsMME through DLS and ζ-potential measurements. Each of the mentioned nanoparticles were suspended in both deionized water and HEPES buffer (25 mM, pH 7.2), with a concentration of 10 μg/mL for particle size and ζ-potential measurements, respectively. The prepared samples were analyzed using the Zetasizer Nano ZS (Malvern Instruments Ltd., Malvern, UK) instrument. Triplicate measurements were conducted for each sample, and the results were presented as mean ± standard deviation (SD). 

The FTIR spectra of the samples were obtained using Invenio R spectrometer (Bruker Optics GmbH, Ettlingen, Germany) equipped with a PA301 photoacoustic detector (Gasera Oy, Turku, Finland) operated at 8 cm^−1^ resolution. For the measurements, the nanoparticle samples were pelletized using centrifugation and dried.

The morphology and size of the nanoparticles in dry state were subsequently investigated using the JEM-1400Plus TEM (Jeol Ltd., Akishima City, Japan). The samples were prepared by dispersing the nanoparticles in EtOH (99.5%) at a concentration of 10 μg/mL using a bath sonicator. Subsequently, a droplet of each sample was deposited onto a TEM grid, left to dry, and placed into the TEM instrument operating with an accelerating voltage of 120 kV. Micrographs were acquired at various magnifications to capture a representative view of the nanoparticles. Particle size measurements were performed using Image J 1.46r software, and the measurements were acquired from a statistically significant number of particles to ensure accuracy. Statistical parameters, such as mean size and standard deviation, were calculated from the collected data by using Microsoft Excel 2021. Further imaging of the appearance of the nanoparticles was conducted using an Apreo S field-emission scanning electron microscopy (SEM) (Thermo Scientific Inc., Eindhoven, The Netherlands). For the imaging, particle dispersions in EtOH were drop-casted on a polished, conductive Si substrate and let to dry. The structural characteristics of the nanoparticles were analyzed with N_2_ and Kr sorption at −196 °C using 3Flex 3500 (Micromeritics Corp., Norcross, GA, USA). The specific surface area (SSA) was calculated using the Brunauer–Emmett–Teller method, and the total pore volume was estimated as the total adsorbed amount at a relative pressure *p*/*p*_0_ = 0.95.

### 2.6. Drug Release from Nanoparticles

To investigate the release of PsMME from the three nanoformulations, 1 mg of each PsMME-loaded nanoparticle was dispersed in 1 mL of three different phosphate-buffered saline (PBS) solutions containing Tween 80 (0.5% *w*/*v*) to provide sink conditions [23] with pH values of 5, 6.8, and 7.4. The amphiphilic property of Tween (as with other surfactants) allows it to solubilize hydrophobic drugs or compounds in aqueous solutions, facilitating their dispersion and release under sink conditions; i.e., enabling the dissolution media to dissolve at least three times the amount of drug in the dosage form. Subsequently, all samples were maintained at 37 °C temperature in a shaker incubator. At various time points (0, 1, 2, 4, 6, 12, 24, 48, and 72 h), the samples were subjected to centrifugation, and the resulting supernatants were diluted with methanol. Finally, the UV-Vis absorption of the samples was measured at a wavelength of 300 nm using the NanoDrop™ 2000c Spectrophotometer (Thermo Scientific Corp. Wilmington, DE) in triplicate. The concentrations were determined utilizing a standard curve. The cumulative drug release was presented graphically [24]. 

To investigate drug release kinetics, in vitro release data were analyzed using various kinetic models, such as zero order, first order, and the Higuchi diffusion model for the first 24 h of the drug release measurements. Additionally, the data were subjected to the Korsmeyer–Peppas equation to assess the drug diffusion mechanism, with a focus on determining the diffusion exponent (*n*). If *n* ≤ 0.49, the release is indicative of a Fickian mechanism, while a range of 0.5 ≤ *n* ≤ 0.8 suggests a non-Fickian mechanism [25].

### 2.7. Cell Culture and Maintenance

The HEK293 cell line was maintained in DMEM, which was supplemented with 10% heat-inactivated FBS, 2 mM L-glutamine, 0.1 mM MEM NEAA, 100 IU/mL penicillin, and 100 μg/mL streptomycin. The cells were incubated at 37 °C in an atmosphere containing 5% CO_2_. Subculturing was performed every 3 days when the cells reached 80–90% confluence [26].

### 2.8. Cytotoxicity of MPDA Nanoparticles before and after Drug Loading 

The Alamar Blue cell proliferation assay was conducted to assess the biocompatibility of PsMME, MPDA, and PsMME-loaded MPDA (MPDA–PsMME). In brief, HEK293 cells (15 × 10^3^ cells/cm^2^) were initially cultured overnight in a 48-well plate [26]. The cell culture medium was then substituted with fresh pre-warmed media containing PsMME (10, 20, 40 μg/mL), corresponding concentrations of MPDA (10, 20, 40 μg/mL), and MPDA–PsMME (20, 40, 80 μg/mL) samples (*n* = 3). Assessment of the nanoparticles’ cytotoxicity was performed after 24 and 48 h of incubation. Following incubation, the Alamar Blue reagent was introduced into each well at a final concentration of 10%. The plate was subsequently incubated at 37 °C for 4 h, allowing resazurin to undergo metabolic conversion. Fluorescence intensity measurements were taken using a spectrophotometer at 570 nm for excitation and a range of 580–600 nm for emission wavelengths utilizing the Thermo Scientific Varioskan Flash multi-plate reader [27,28,29]. The data in each time point were normalized by their corresponding control, and the percentage of cell viability was calculated according to the following Equation (3) by using relative fluorescence units (RFU) [30,31].

(3) $$Cell\;viability\%=\frac{Experimental\;RFU\;with\;chemical\;compound-background\;RFU}{Untreated\;cell\;control\;RFU\;value-background\;RFU}*100$$

### 2.9. Photostability, Gas Chromatography, and Gas Chromatography–Mass Spectrometry Analysis

Sample preparation for the photostability test involved dissolving a specified amount of each loaded nanoparticle in 2 mL of deionized water. Subsequently, the samples were dispersed using a sonicator and placed in contact with a 125 W UV-light source for a duration of 30 min. To account for potential effects of drug release on the gas chromatography with a flame ionization detector (GC-FID) (Clarus 500, PerkinElmer, Inc., Waltham, MA, USA) and GC-MS analysis, control samples that were not exposed to UV light were subjected to the same stirring conditions as the other samples. Afterwards, the samples were centrifuged at 13k rpm for 15 min to separate the water components. The resulting supernatant was carefully removed, and the sediment was reconstituted in 1 mL of methanol. It was then sonicated for 1 h to facilitate the release of PsMME from the nanoparticles. Afterward, the samples underwent another round of centrifugation, lasting 20 min at 18,000 rpm and 10 °C. Finally, the supernatants were vacuum dried for subsequent GC-FID and GC-MS analysis.

For GC-FID and GC-MS analysis a precise quantity of each sample was dissolved in 1 mL of HPLC-grade acetone. From each sample, 0.5 mL was aliquoted into a test tube, to which 2 mL of an internal standard (IS) solution containing 0.02 mg of each of the pure compounds (>95%), namely n-heneicosanoic acid, betulinol, cholesteryl heptadecanoate, and 1,3-dipalmitoyl-2-oleyl glycerol, was added. The solvent was then removed under nitrogen gas at 40 °C and further desiccated in a vacuum desiccator for 20 min. Following solvent removal, the samples were treated with a silylation reagent consisting of 0.12 mL pyridine/BSTFA/TMCS [1:4:1] mixture. These treated samples were heated in an oven at 70 °C for 45 min. Quantitative analysis was based on triplicate measurements of the same formulation. The samples were analyzed with GC-FID, using two long columns in parallel (HP-1 and HP-5). The detailed GC-FID conditions can be found in Smeds et al. [32]. The GC-MS instrument was an Agilent Technologies 7890-5975 GC-MSD instrument (Agilent Technologies, Inc., Wilmington, NC, USA) with a HP-5 column (30 m × 250 μm × 0.25 μm).

Identification of PsMME was achieved by comparing the characteristic mass spectra with reference databases (NIST12/Wiley11th and a database developed at the Laboratory of Natural Materials Technology at Åbo Akademi University). For calculations, n-heneicosanoic acid (21:0) was used as the internal standard for PsMME. The recovery% of PsMME was calculated by using Equation (4).

(4) $$Recovery\%=\frac{Amount\left(mean\;of\;three\;experiment\right)\;of\;residual\;PsMME\;in\;the\;test\;groups}{Amount\left(mean\;of\;three\;experiment\right)\;of\;residual\;PsMME\;in\;the\;control\;groups}*100$$

### 2.10. Statistical Analysis

Statistical analysis was performed utilizing GraphPad Prism 6 software, employing a two-way ANOVA followed by Tukey’s multiple comparisons test. The presented data are expressed as “mean ± standard deviation” values. 

## 3. Results and Discussion 

### 3.1. Synthesis and Characterization of Nanoformulations

In this study, we synthesized three distinct dark nanoformulations of PDA–PEI–MSNs, MPDA, and TCPSi, with the aim of assessing and comparing their effectiveness in enhancing the photostability of loaded PsMME. The MPDA and TCPSi nanoparticles possess a dark coloration, whereas in the case of the PDA–PEI–MSN descriptor, the term “dark” is utilized to denote the modified color of the mesoporous silica nanoparticles due to the presence of a polydopamine coating [33].The sizes of the nanoparticles (Table 1) obtained by DLS measurements were larger than those obtained by TEM, which is to be expected, as DLS measures the overall hydrodynamic diameter including the solvation layer around the nanoparticles. This layer can significantly contribute to the apparent size in a solution, making particles appear larger than their dry-state size observed in TEM [34]. Moreover, porosity affects the size difference between DLS and TEM even more prominently, as shown in one of our previous studies [33]. 

**Figure 1:**
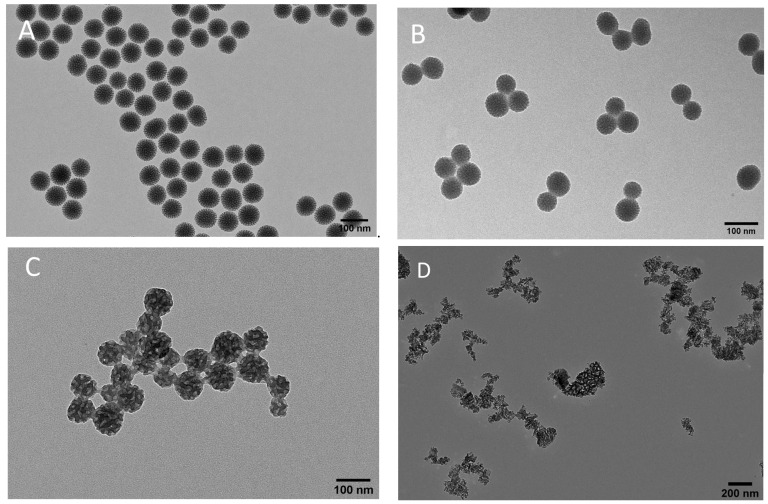
TEM images of MSNs (**A**), PDA–PEI–MSNs (**B**), MPDA (**C**), and TCPSi (**D**) nanoparticles.

A key modification involved grafting PEI onto the MSNs, thereby facilitating the subsequent PDA coating by introducing amine groups to the surface. The hydrodynamic diameters (Z-average) for MSNs, PEI-MSNs, and PDA–PEI–MSNs were determined to be 96.7 ± 1.21 nm, 118.8 ± 1.70 nm, and 147.5 ± 1.1 nm, respectively. The notably increased size of PDA–PEI–MSNs further corroborated the formation of a polymerized layer of dopamine on the PEI-MSN surface. ζ-potential analysis revealed a shift in the MSN net surface charge from −27.8 ± 1.0 mV to +32.9 ± 1.3 mV under identical HEPES buffer conditions (25 mM, pH 7.2) after the grafting of PEI. This change can be attributed to the introduction of a high abundance of amine groups on the MSN surface. Following PDA coating of PEI-MSNs, the ζ-potential shifted to negative again (−31.3 ± 1.8 mV) due to the presence of hydroxyl groups of the formed polydopamine layer (Table 1). Microscopic observation by TEM showed spherical nanoparticles with dendritic pore structure for MSNs, PEI-MSNs, and PDA–PEI–MSNs, with sizes centered at 73.6 ± 1.9 nm, 62.8 ± 1.4 nm, and 93.95 ± 4.49 nm, respectively (Figure 1A,B), based on image analysis (Image J).

Notably, the PDA coating significantly increased the size of PEI-MSNs. The conducted TEM analysis of MPDA nanoparticles with Image J indicated a size of 78.2 ± 3.0 nm with a spherical porous structure (Figure 1C). A ζ-potential of −10 ± 0.1 mV was measured as the net surface charge of MPDA nanoparticles at pH 7.2 due to the availability of phenolic hydroxyl groups (Table 1). Owing to the top-down fabrication method, TEM revealed TCPSi nanoparticles of varying sizes and shapes, with considerably larger average size of about 180 nm (Figure 1D), corresponding to the fracture plane-creating etching profile. The further change in ζ-potential of the nanoparticles after loading of PsMME, demonstrated in Table 1, indicates successful loading of the PsMME.

Comparing the FTIR spectra of the dry, bare nanoparticles to the PsMME-loaded nanoparticles, Figure S3 presents the main features of the PsMME overlaid on the spectra of the empty particles. The prominent features of PsMME, such as the OH-stretching vibrations near 3350 cm^−1^ as well as the ring-related stretching and deformation vibrations at 1605 and 1505 cm^−1^, respectively, are visible across all nanoparticle types [35]. Similarly, the typical features of the nanoparticles can be observed in all the nanoformulations. The broad absorbance band ascribed to Si–O–Si stretching vibrations of the silica matrix at 1100 cm^−1^ as well as the effects of the PEI–PDA in the modification in the aliphatic C–H stretching region at 2855 and 2925 cm^−1^ can be observed convoluted over the PsMME features in the corresponding regions [36]. The broad absorbance features of MPDA are also readily observed in MPDA–PsMME spectra [37]. As with the silica, the main absorbance bands of TCPSi, the Si–O and Si–C related vibrations near 1050–1100 cm^−1^ are highly visible in the TCPSi–PsMME nanoformulation [38].

**Figure 2:**
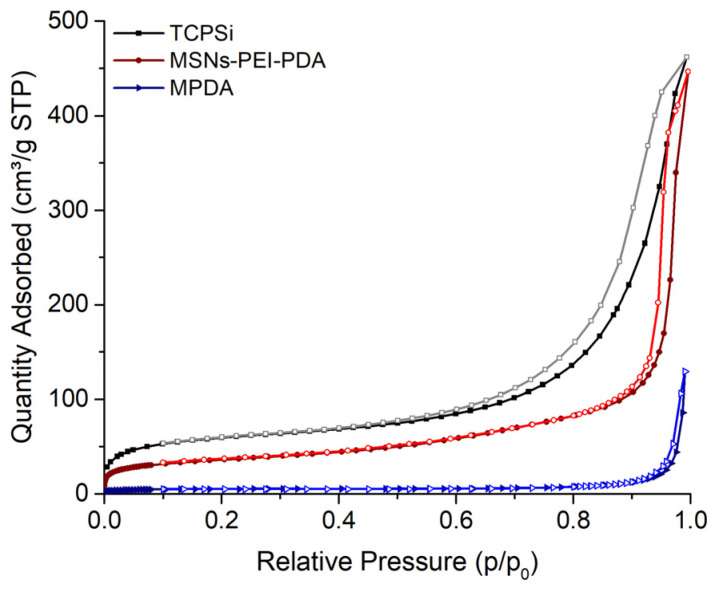
Nitrogen adsorption–desorption isotherms of TCPSi, PDA–PEI–MSNs, and MPDA nanoparticles.

**Figure 3:**
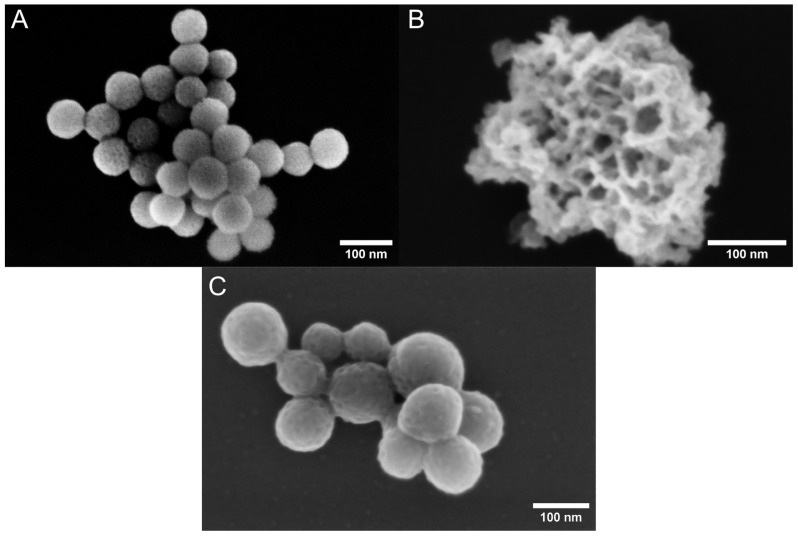
Secondary electron micrographs of PDA–PEI–MSNs (**A**), TCPSi (**B**), and MPDA (**C**) nanoparticles acquired with SEM using an in-column detector at 2 kV accelerating voltage.

The porous characteristics of the nanoparticles were further analyzed with nitrogen sorption. The adsorption/desorption isotherms are shown in Figure 2, revealing clear differences between the PDA–PEI–MSNs, TCPSi, and MPDA nanoparticles. The uncoated TCPSi nanoparticles show the largest specific surface area (SSA), while the coated MSNs and MPDA nanoparticles indicate considerably lower SSAs, owing to the added surface coatings. For the MPDA, the observed SSA was also verified with krypton adsorption. As the nanoparticles did not show a clear plateau at high relative pressures, despite their isotherms indicating a mesoporous structure, the pore volume can only be estimated, as intraparticle voids may also contribute to the calculated volumes. In this manner, the total pore volumes calculated from the desorption branch of the isotherms suggested the particles to have 0.59 cm^3^/g, 0.66 cm^3^/g, and 0.16 cm^3^/g internal volume for PDA–PEI–MSNs, TCPSi, and MPDA, respectively. Low volumes and areas observed with the PDA–PEI–MSNs and MPDA particles were likely due to the combination of coating on the mesoporous silica particles and the non-native dry state of the nanoparticles during the analysis. The secondary electron micrographs that were taken using low accelerating voltage are shown in Figure 3. These micrographs indicate that the TCPSi have open pores, while with the PDA–PEI–MSNs and MPDA nanoparticles, the outlines of the pores can be observed, but these appear blocked due to the presence of the dried coating. Here, measurements in dry state (TEM imaging and nitrogen sorption) may be causing this artefact, considering that drug loading is carried out in solution, whereby the coating would not be dried and would thus not cause pore blocking. PsMME with a low molecular weight of 226.28 g/mol would fit readily into small-sized mesopores. 

### 3.2. Drug Incorporation Capacity and Efficiency Comparison between Nanoparticles

Among the critical parameters governing the effectiveness of these systems, drug loading degree (DL%) and encapsulation efficiency (EE%) stand out as crucial factors that dictate the therapeutic potential of nanoparticles. Aiming at comparable loading degrees that would also be compatible with photoprotection measurements, we loaded PDA–PEI–MSNs, TCPSi, and MPDA with PsMME via the solvent evaporation method. Employing this loading procedure, the PDA–PEI–MSNs exhibited the highest values for DL% (19.53 ± 0.6), EE% (95.57 ± 2.9), and drug content (0.16 ± 0.16 mg/mg) as *m*_drug_/*m*_drug+carrier_ (Table 2). The superior DL% and EE% observed for PDA–PEI–MSNs, in comparison with the other two nanoparticle types, can be attributed to their well-defined pore structures boasting uniform pore sizes [39]. This unique characteristic in general facilitates effective drug loading and encapsulation. Furthermore, the synthesis methods of MSNs compared with those of MPDA and TCPSi can influence the drug loading capabilities of the nanoparticles. That is, the controlled synthesis of MSNs involves templating processes that create precisely engineered structures for homogeneous drug loading throughout the matrix. In contrast, the synthesis of MPDA through dopamine polymerization might result in less uniform and less predictable structures, potentially affecting drug loading capability and homogeneity [6,40]. 

### 3.3. Drug Release from Nanoparticles 

**Figure 4:**
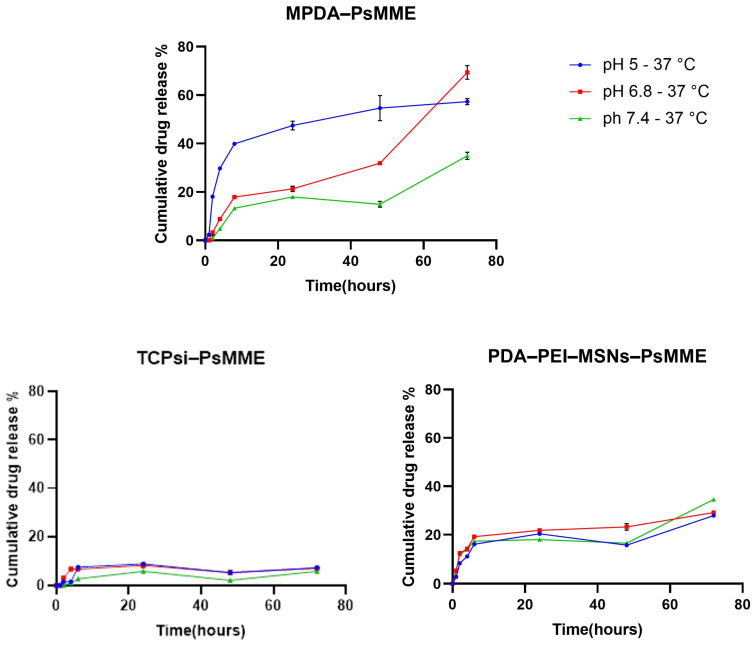
In vitro PsMME release from MPDA, TCPSi, and PDA–PEI–MSNs at different pH values: acetate buffer (5.5), phosphate buffer (6.8), and PBS (7.4) at 37 °C (*n* = 3).

Nanoparticles can be used to extend the duration of drug release, which gives them certain advantages over immediate-release formulations, such as the potential for reducing dosing frequency and maintaining consistent drug concentration over time [41,42]. In this study, the PsMME release from the three nanoparticle formulations PDA–PEI–MSNs, TCPSi, and MPDA was carried out in acetate buffer, phosphate buffer, and PBS at the three different pH conditions of 5.5, 6.8, and 7.4, respectively, at 37 °C for 72 h (Figure 4), corresponding to endosomal, intratumoral/inflamed tissue, and extracellular conditions. According to statistical analysis, there are significant differences in drug release between the three different pH conditions (pH 5.5, 6.8, and 7.4) for each of the three nanoparticles (**** *p* < 0.0001). This may be owing to polydopamine (in PDA–PEI–MSNs and MPDA) being sensitive to low pH, i.e., being more soluble under acidic conditions, due to the protonation of its functional groups [43]. Therefore, a higher percentage of PsMME accumulative release can be observed at lower pH [44,45]. Since the other components (MSN, TCPSi) are not pH-responsive, the slight differences in release behavior under different pH conditions can most likely be attributed to the pH-dependent solubility of the drug molecule itself. Overall, the drug release profiles of the three nanoformulations show a sustainable release pattern over 72 h, which is an indication of incorporation of PsMME in the pores of the nanoparticles. MPDA showed the highest drug release fraction of 69.33 ± 1.62% after 72 h at pH 6.8 compared to the other nanoparticles and release conditions. 

On the other hand, TCPSi, with a cumulative drug release of 5.8 ± 0.2% at pH 7.4 at the same timepoint, showed the lowest fraction of drug release. The release of the drug from the three nanoformulations was observed to adhere to the Higuchi diffusion model, providing the optimal fit and indicating diffusion as the primary release mechanism. However, an exception was noted in the MPDA-PsMME formulation at pH 6.8, which conformed to the zero-order model. A zero-order release would not be expected from such a system, so the reason for this observation may be the few data points at hand. The determined exponent (*n*) value for the majority of formulations was observed to be ≤0.5, indicating that the release of PsMME from these formulations was driven by Fickian diffusion (Tables S1–S3 in the Supplementary Data). 

### 3.4. Photostability Study 

**Figure 5:**
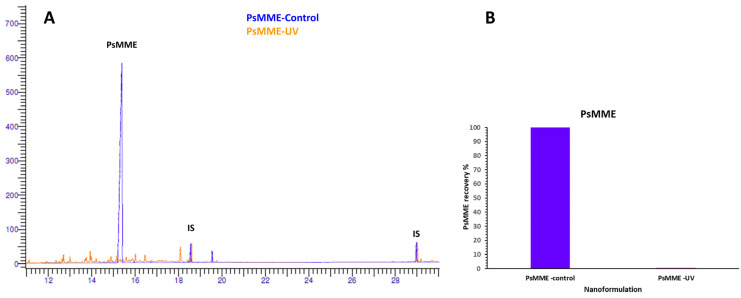
(**A**) The superimposition of GC–FID chromatograms of PsMME–control (before UV exposure) and PsMME–UV (after UV exposure); IS—internal standard. (**B**) The recovery percentage of PsMME after exposure to UV light.

**Figure 6:**
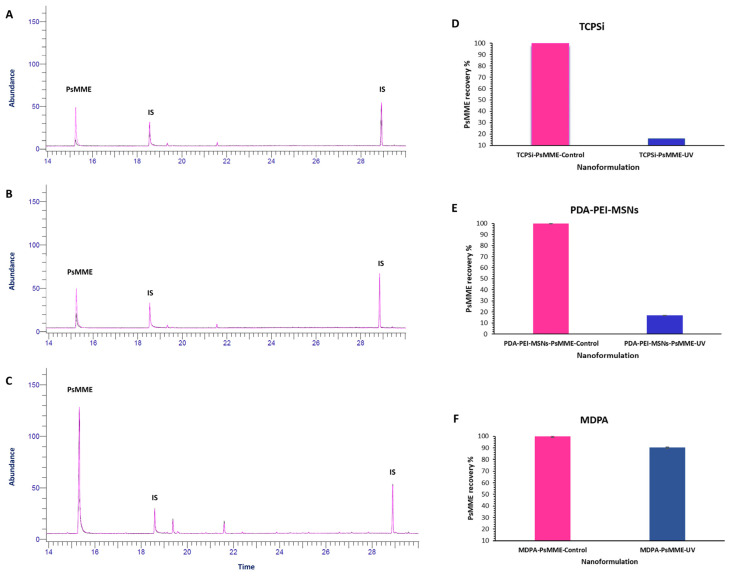
Left: the superimposition of GC–FID chromatograms of the controls and the test samples: TCPSi–PsMME (**A**), PDA–PEI–MSNs–PsMME (**B**), and MPDA–PsMME (**C**). Right: the recovery percentage of PsMME for the TCPSi (**D**), PDA–PEI–MSNs (**E**), and MPDA (**F**) formulations. The control and test samples are represented in pink and blue colors, respectively.

To determine and compare the effect of our dark nanoparticles on PsMME photostability, GC-FID analysis was conducted on the three nanoformulations of PDA–PEI–MSN–PsMME, TCPsi–PsMME, and MPDA–PsMME before (controls) and after they were exposed to the UV light. The aim was to measure the relative residual PsMME content (recovery%) within the nanoparticles after the UV treatment. Simultaneously, the corresponding three control nanoformulations were maintained in a stirred state to observe any potential influence of the preparation process on the results. Afterward, the specific impact of UV exposure on the PsMME content in these formulations was calculated. The impact of the UV light exposure on PsMME was assessed through GC–FID, revealing a significant degradation of more than 90% without any nanoparticles (Figure 5). PDA–PEI–MSN and TCPSi formulations exhibited protection against the UV-light induced degradation, with 16.75% (Figure 6B,E) and 15.88% (Figure 6A,D) recoveries, respectively, following a 30-min UV light exposure. Intriguingly, MDPA demonstrated remarkable protective properties, preserving 90.5% of the loaded PsMME under the same conditions (Figure 6C,F). The percentage of recovery was calculated using Equation (4).

### 3.5. Cellular Viability of MPDA–PsMME

**Figure 7:**
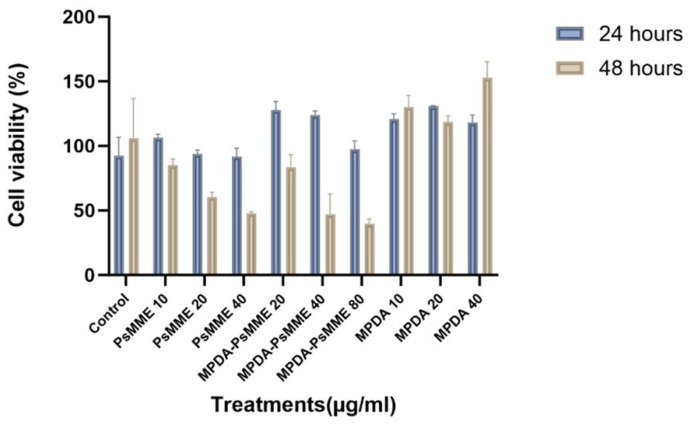
Cytocompatibility of PsMME and MPDA–PsMME incubated with HEK293 cells for 24 and 48 h.

Recognizing the most promising photoprotective characteristics of MPDA nanoparticles for PsMME among the ones studied, as evidenced by their superior 90.5% protective effect against UV light, we further investigated the cytocompatibility of this specific nanoformulation, paving the way for future pharmaceutical studies. To evaluate the cytocompatibility of the MPDA–PsMME nanoformulation, we conducted a cell viability assessment by examining its influence on cell proliferation using the Alamar Blue assay. The HEK-293 cells were selected as a model cell line due to their extensive utilization in pharmaceutical research for cytocompatibility assessment. This choice is rooted in their specific attributes, including a high proliferation rate and their ability to adapt to various culture media [46]. The HEK293 cells were exposed to distinct concentrations of PsMME (10, 20, 40 μg/mL) alongside corresponding concentrations of MPDA (5, 10, 20 μg/mL) and MPDA–PsMME (20, 40, 80 μg/mL) individually (*n* = 3) for both 24 and 48 h, as illustrated in Figure 7. Statistical analysis was carried out for the treated HEK293 cells utilizing a two-way ANOVA, revealing a significant variance in cell viability between the two time points (*p* < 0.0001). Tukey’s multiple comparisons test indicated that there were no notable differences in viability between HEK293 cell samples treated with MPDA–PsMME 20 μg/mL (** *p* = 0.0037), MPDA–PsMME 40 μg/mL (* *p* = 0.0138), MPDA 10 μg/mL (* *p* = 0.0389), and MPDA 20 μg/mL (** *p* = 0.0012) in comparison with the control sample after 24 h. However, after 48 h, there were significant differences in cell viability for PsMME 20 μg/mL, PsMME 40 μg/mL, MPDA–PsMME 40 μg/mL, MPDA–PsMME 80 μg/mL, and MPDA 40 μg/mL compared to the control sample (**** *p* < 0.0001). These findings suggest that MPDA itself might exhibit a stimulatory effect on the proliferation of HEK293 cells at lower concentrations. Therefore, it can be inferred that MPDA nanocarriers are safe for in vitro applications at concentrations up to 40 μg/mL. However, elevating the PsMME concentration beyond 10 μg/mL seems to lead to decreased cell proliferation after 24 h of treatment, suggesting a toxic effect of PsMME when exposed to cells at doses exceeding 10 μg/mL for durations longer than 24 h.

## 4. Conclusions

PsMME’s pronounced photosensitivity poses a significant challenge, particularly in pharmaceutical context. This study addresses this issue by utilizing dark porous nanoparticles as photoprotective carriers. PsMME was loaded into three distinct nanoparticles—PDA–PEI–MSNs, TCPSi, and MPDA—with the aim of identifying the most effective protector against UV light. Following UV irradiation of PsMEE–loaded nanocarriers, PsMME recovery percentage was evaluated through GC–FID analysis. Despite PDA–PEI–MSNs being superior in terms of drug loading degree compared to the other nanoparticles, MPDA stands out with an exceptional 90% recovery rate for PsMME, which positions MPDA as the optimal photoprotective carrier. In contrast, TCPSi and PDA–PEI–MSNs exhibited significantly lower levels of protection, with recovery rates of 16.75% and 15.88%, respectively. This research underscores the potential of MPDA nanocarriers as promising candidates for protecting PsMME and other photosensitive drug compounds against UV light, paving the way for enhanced utilization in various applications, particularly within pharmaceuticals. In dosage form design, nanoformulation of PsMME facilitates its further formulation compared to the free drug molecule, as this course of action evades the consideration of the physicochemical characteristics, such as photolability, of PsMME itself in the following steps. Given that PsMME serves as a model for photolabile drugs in our study, the same nanoformulation approach can be extended to other photolabile drugs as well. 

## Figures and Tables

**Table 1:** Average sizes, PDI, ζ-potential (in 25 mM HEPES buffer, pH 7.2), and specific surface area of the studied nanoparticles.

Name of NPs	Hydrodynamic Diameter (nm)	PDI	ζ-Potential (mV)	TEM Size (nm)	Specific Surface Area (m^2^/g)
MSNs	96.7 ± 1.21	0.07 ± 0.00	−27.8 ± 1.0	73.6 ± 1.9	-
PEI–MSNs	118.8 ± 1.70	0.08 ± 0.02	+32.9 ± 1.3	62.8 ± 14	-
PDA–PEI–MSNs	147.5 ± 1.10	0.07 ± 0.03	−31.3 ± 1.8	93.9 ± 4.5	129 ± 2
PDA–PEI-MSNs–PsMME	157 ± 1.82	0.83 ± 0.00	−11.3 + 0.01	-	-
TCPSi	213.3 ± 0.12	0.12 ± 0.01	−18.1 ± 1.8	180.6 ± 6.5	218 ± 2
TCPSi–PsMME	447 ± 1.6	0.239 ± 0.00	−14 ± 0.00	-	-
MPDA	180.6 ± 12.49	0.35 ± 0.06	−10.1 ± 0.1	78.2 ± 3.0	26 ± 1
MPDA–PsMME	124 ± 2.34	0.536 ± 0.12	−9.37 ± 0.03	-	-

**Table 2:** Drug loading degree (DL%), encapsulation efficiency (EE%), and drug content of PDA–PEI–MSNs, TCPSi, and MPDA nanoparticles.

Name of NPs	PsMME/NPs (wt%)	DL (%) ± SD	EE (%) ± SD	Drug Content (mg/mg)
PDA–PEI–MSNs	20%	19.53 ± 0.6	95.57 ± 2.9	0.16 ± 0.16
TCPSi	20%	11.43 ± 0.5	55.95 ± 2.5	0.100 ± 0.10
MPDA	20%	6.89 ± 0.7	34.49 ± 3.4	0.063 ± 0.063

## Data Availability

The data presented in this study are available within the article and Supplementary Material.

## Supplementary Materials

The following supporting information can be downloaded at: https://www.mdpi.com/article/10.3390/pharmaceutics16020276/s1. Figure S1: GC-MS chromatogram of PsMME (peak number 2) and the area percent report (purity: 98.6%). Figure S2: GC-MS spectra of MPDA–PsMME before (A) and after (B) UV exposure. (C) Mass spectra for silylated PsMME. Table S1: Release kinetics of PEI–PDA–MSN–PsMME. Table S2: Release kinetics of MPDA–PsMME. Table S3: Release kinetics of TCPSi–PsMME. Figure S3: FT-IR spectra of (a) PsMME-MSNs, (b) PsMME-MPDA, and (c) PsMME-TCPSi nanoformulations accompanied by PsMME and plain NPs as reference.
