# Supplementary material for: Investigating the Effectiveness of Different Porous Nanoparticles as Drug Carriers for Retaining the Photostability of Pinosylvin Derivative
Source: Pharmaceutics. 2024 Feb 15;16(2):276. doi: 10.3390/pharmaceutics16020276 (PMC10892027; doi:10.3390/pharmaceutics16020276)
Supplement: Supplementary file 1 [file pharmaceutics-16-00276-s001.zip › pharmaceutics-2725792-supplementary.pdf]

Supplementary Material for article:

Investigating the effectiveness of different porous nanoparticles as drug carriers for retaining the photostability of pinosylvin derivative

Fadak Howaili [1], Atefeh Saadabadi [1,2,3], Ermei Mäkilä [4], Ekaterina Korotkova [5], Patrik C. Eklund [2], Outi M. H. Salo-Ahen [1,3], Jessica M. Rosenholm [1]

[1] Pharmaceutical Sciences Laboratory, Faculty of Science and Engineering, Åbo Akademi University, 20500 Turku, Finland
[2] Laboratory of Molecular Science and Engineering, Faculty of Science and Engineering, Åbo Akademi University, 20500 Turku, Finland
[3] Structural Bioinformatics Laboratory, Faculty of Science and Engineering, Åbo Akademi University, 20500 Turku, Finland
[4] Laboratory of Industrial Physics, Department of Physics and Astronomy, University of Turku, 20014 Turku, Finland
[5] Laboratory of Natural Materials Technology, Faculty of Natural Sciences and Technology, Åbo Akademi University, 20500 Turku, Finland

Area Percent Report

```
Data Path : C:\msdchem\2\data\Ati\Natural compounds\
Data File : PsMME-Pure-no silylation.D
Acq On    : 29 Mar 2019  12:18
Operator  : Ati
Sample    :
Misc      :
ALS Vial  : 19   Sample Multiplier: 1

Integration Parameters: autoint1.e
Integrator: ChemStation

Method    : C:\MSDCHEM\2\METHODS\default.m
Title     :

Signal     : EIC TIC: PsMME-Pure-no silylation.D\data.ms
```

peak #	R.T. min	first scan	max scan	last scan	PK TY	peak height	corr. area	corr. % max.	% of total
1	21.656	2379	2399	2404	BV	181065	3187848	1.41%	1.393%
2	21.758	2404	2413	2466	VV	12083217	225643927	100.00%	98.607%

Sum of corrected areas: 228831775

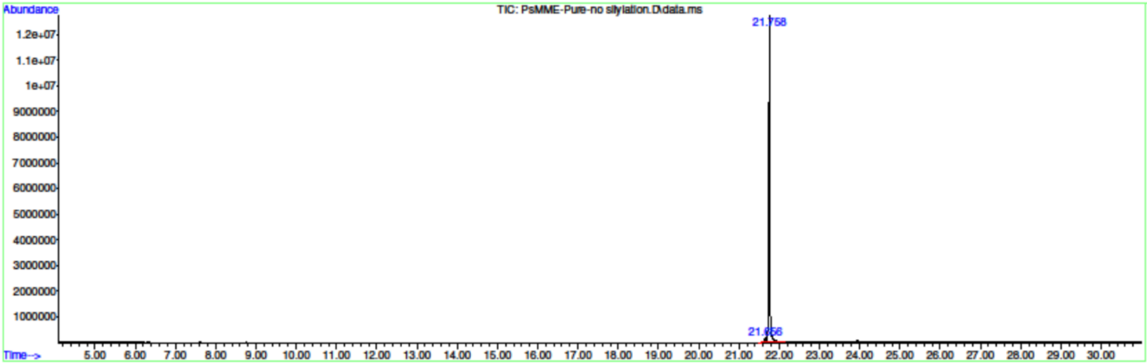

Figure S1. GC-MS chromatogram of PsMME (peak number 2) and the area percent report (purity: 98.6 %).

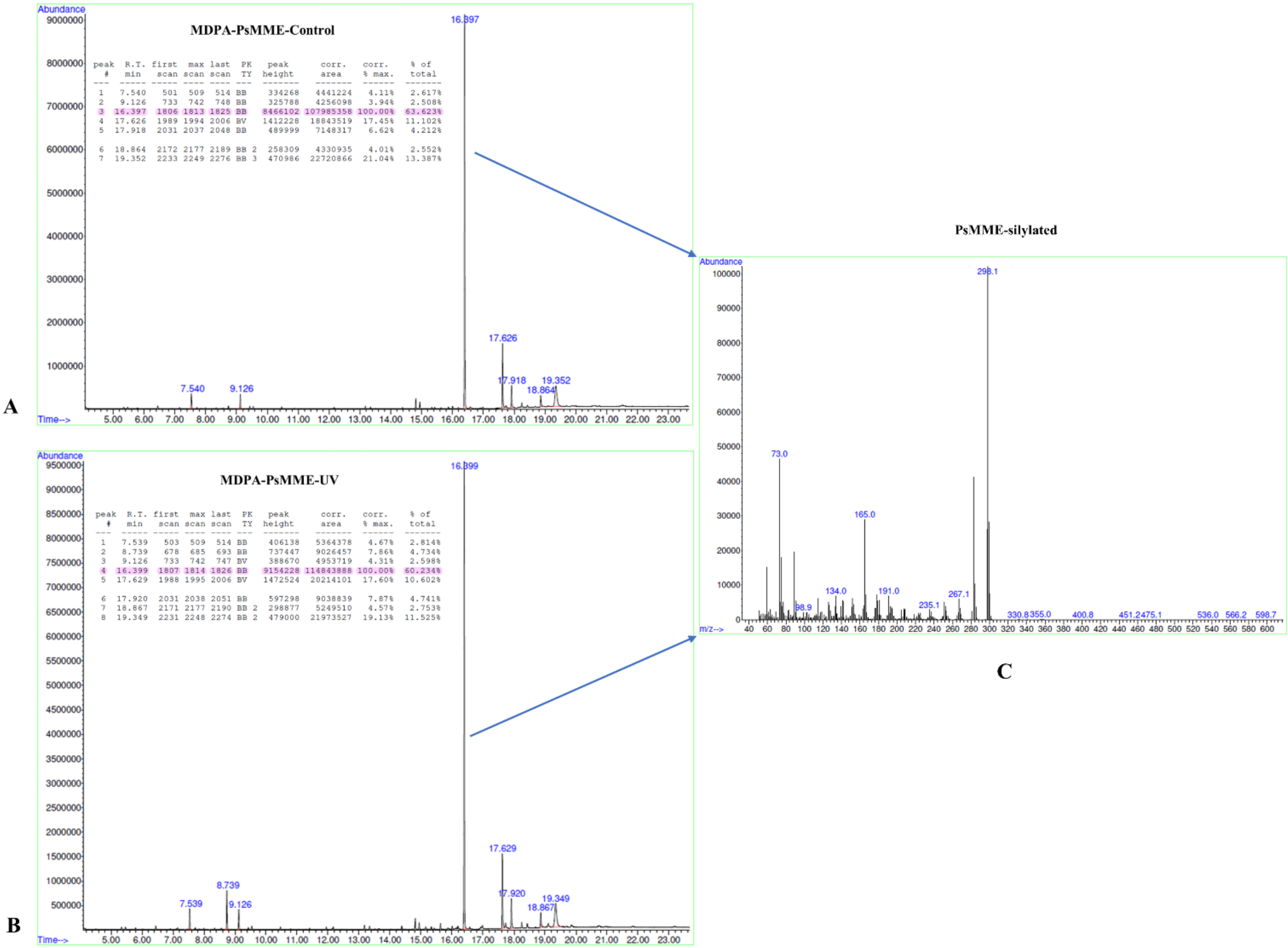

Figure S2. GC-MS spectra of MPDA-PsMME before (A) and after (B) UV exposure. (C) Mass spectra for silylated PsMME.

Table S1. Release kinetics analysis of PEI-PDA-MSN-PsMME.

PEI-PDA-MSN-PsMME	Zero order		First order		Higuchi diffusion		Korsmeyer-Peppas	
	R^2	K	R^2	K	R^2	K	R^2	n
**pH: 5**	0.8344	0.5969	0.8490	0.0069	0.9128	4.0519	0,9497	0,3537
**pH: 6.8**	0.7639	0.5147	0.7791	0.0060	0.8560	3.5789	0,9272	0,2269
**pH: 7.4**	0.6694	0.3471	0.6791	0.0040	0.7803	2.5110	0,8830	0,1579

Table S2. Release kinetics analysis of MPDA-PsMME.

MPDA-PMME	Zero order		First order		Higuchi diffusion		Korsmeyer-Peppas	
	R^2	K	R^2	K	R^2	K	R^2	n
**pH: 5**	0.8675	0.4652	0.9075	0.0081	0.9354	4.9794	0,9444	0,3758
**pH: 6.8**	0.9581	0.8045	0.9261	0.0138	0.9252	7.7115	0,9204	0,7350
**pH: 7.4**	0.8933	0.3762	0.8963	0.0046	0.8964	3.7477	0,9186	1,1453

Table S3. Release kinetics analysis of TCPSi –PsMME.

TCPSi-PsMME	Zero order		First order		Higuchi diffusion		Korsmeyer-Peppas	
	R^2	K	R^2	K	R^2	K	R^2	n
**pH: 5**	0.7773	0.2986	0.7802	0.0031	0.8249	2.0961	0,8364	0,7813
**pH: 6.8**	0.7171	0.1562	0.7221	0.0016	0.7866	1.1334	0.9575	0.1185
**pH: 7.4**	0.9638	0.2129	0.9655	0.0022	0.9767	1.5233	0.7339	0.7537

R^2 = coefficient of determination, n = release exponent, k= kinetic constant

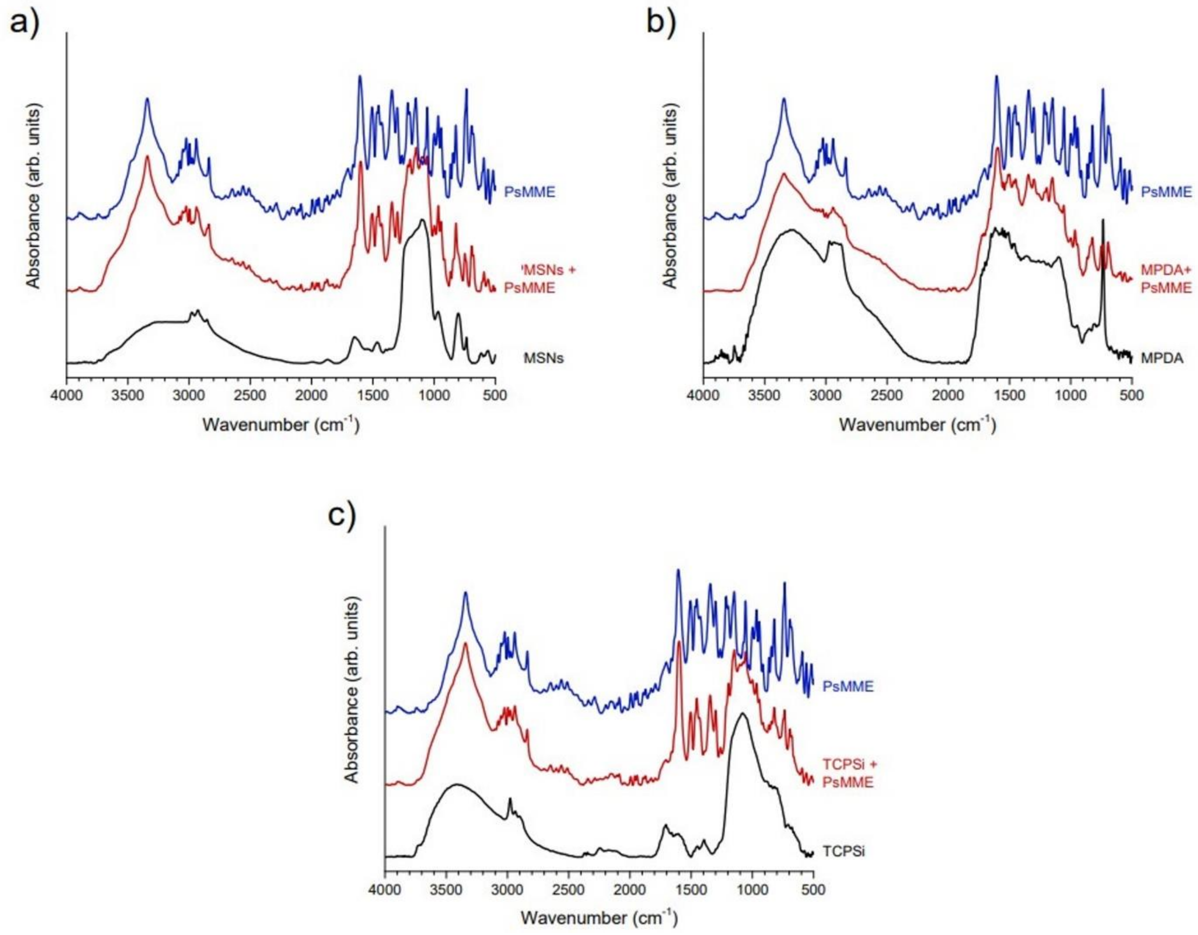

Figure S3. FT-IR spectra of (a) PsMME-MSNs, (b) PsMME-MPDA, and (c) PsMME-TCPSi nanoformulations accompanied by PsMME and plain NPs as reference.
